# Identification of candidate genes involved in Witches’ broom disease resistance in a segregating mapping population of *Theobroma cacao* L. in Brazil

**DOI:** 10.1186/s12864-016-2415-x

**Published:** 2016-02-11

**Authors:** Stefan Royaert, Johannes Jansen, Daniela Viana da Silva, Samuel Martins de Jesus Branco, Donald S. Livingstone, Guiliana Mustiga, Jean-Philippe Marelli, Ioná Santos Araújo, Ronan Xavier Corrêa, Juan Carlos Motamayor

**Affiliations:** Mars Center for Cocoa Science, CP 55, Itajuípe, BA CEP 45.630-000 Brazil; Biometris, Wageningen University and Research Centre, P.O. Box 100, 6700 AC Wageningen, The Netherlands; Departamento de Ciências Biológicas, Universidade Estadual de Santa Cruz, Rodovia Ilhéus-Itabuna, Km 16, Bairro Salobrinho, Ilhéus, BA CEP 45.662-900 Brazil; Mars, Incorporated, 13601 Old Cutler Road, Miami, FL 33158 USA; Departamento de Ciências Vegetais, Universidade Federal Rural do Semi-Arido, BR 110 - Km 47, Bairro Pres. Costa e Silva, Mossoró, RN CEP 59.625-900 Brazil

**Keywords:** *Theobroma cacao* L, SNP, Genetic linkage map, Witches’ broom disease, Marker-trait associations, Candidate genes

## Abstract

**Background:**

Witches’ broom disease (WBD) caused by the fungus *Moniliophthora perniciosa* is responsible for considerable economic losses for cacao producers. One of the ways to combat WBD is to plant resistant cultivars. Resistance may be governed by a few genetic factors, mainly found in wild germplasm.

**Results:**

We developed a dense genetic linkage map with a length of 852.8 cM that contains 3,526 SNPs and is based on the MP01 mapping population, which counts 459 trees from a cross between the resistant ‘TSH 1188’ and the tolerant ‘CCN 51’ at the Mars Center for Cocoa Science in Barro Preto, Bahia, Brazil. Seven quantitative trait loci (QTL) that are associated with WBD were identified on five different chromosomes using a multi-trait QTL analysis for outbreeders. Phasing of the haplotypes at the major QTL region on chromosome IX on a diversity panel of genotypes clearly indicates that the major resistance locus comes from a well-known source of WBD resistance, the clone ‘SCAVINA 6’. Various potential candidate genes identified within all QTL may be involved in different steps leading to disease resistance. Preliminary expression data indicate that at least three of these candidate genes may play a role during the first 12 h after infection, with clear differences between ‘CCN 51’ and ‘TSH 1188’.

**Conclusions:**

We combined the information from a large mapping population with very distinct parents that segregate for WBD, a dense set of mapped markers, rigorous phenotyping capabilities and the availability of a sequenced genome to identify several genomic regions that are involved in WBD resistance. We also identified a novel source of resistance that most likely comes from the ‘CCN 51’ parent. Thanks to the large population size of the MP01 population, we were able to pick up QTL and markers with relatively small effects that can contribute to the creation and selection of more tolerant/resistant plant material.

**Electronic supplementary material:**

The online version of this article (doi:10.1186/s12864-016-2415-x) contains supplementary material, which is available to authorized users.

## Background

*Theobroma cacao* L. (cacao), which is native to the Amazonian basin of South America [1, 2], is a perennial tree that belongs to the Malvaceae *sensu lato* [3] and has a diploid chromosome number of 2n = 2x = 20 [4]. Recently, two cacao genomes have been published. ‘B97-61/B2’, a member of the Criollo genetic group, has an estimated genome size of 409 Mbp [5] whereas the ‘Matina 1–6’ genome, a member of the Amelonado genetic group and more representative of the cacao that is cultivated worldwide, has an estimated genome size of 445 Mbp [6].

Witches’ broom disease (WBD), which causes significant losses, is only present in South America and in the Caribbean [7] and was first reported in 1785 by the explorer Alexandre Rodrigues in the Brazilian Amazon basin [8]. The fungus *Moniliophthora perniciosa* (Stahel) Aime & Phillips-Mora is a basidiomycete and produces basidiocarps, which emerge from dry brooms in the form of mushroom-like structures. Direct production losses are due to infected mature pods and flower cushions causing parthenocarpic fruits, thereby reducing significantly the potential production [9].

A major source of resistance was found in the clones ‘SCAVINA 6’ (‘SCA 6’) and ‘SCAVINA 12’ (‘SCA 12’) [10] and in two other clones, ‘CAB 208’ and ‘CAB 214’, which are genetically different from ‘SCA 6’ [11]. QTL associated with resistance to vegetative brooms (VB) have been identified in three mapping populations. The first population was developed by selfing one tree, ‘TSH 516’, which was obtained from a cross between the resistant ‘SCA 6’ and the susceptible ‘ICS 1’. A major QTL was identified on chromosome IX [12–14], whereas a minor QTL was identified on chromosome I [12]. The two other populations consist of the progenies of a cross between the susceptible ‘ICS 39’ and the resistant ‘CAB 208’ and of a cross between ‘ICS 39’ and the resistant ‘CAB 214’ [11]. One QTL was identified on chromosome IX and is located at a slightly different position than the QTL that was identified in the F_2_ mapping population of ‘SCA 6’ × ‘ICS 1’. Two other QTL were identified on chromosomes IV and VIII [11].

One of the main requirements of an efficient marker-assisted selection (MAS) program is to have reliable markers associated with traits of interest. The availability of dense genetic linkage maps is one of the prerequisites. The first linkage map in cacao, which contained 202 markers, was published in 1995 [15]. This reference map was improved over time through the addition of more trees and markers [16–19]. The most recent consensus map, which also has the highest marker density, combined the data of the cross ‘UPA402’ × ‘UF676’ and of the F_2_ mapping population resulting from selfing a single tree of the cross ‘SCA 6’ × ‘ICS 1’. This map contains 1,262 co-segregating markers and has a length of 733.6 cM [19].

Here, we describe the development of a dense genetic linkage map using single nucleotide polymorphisms (SNP) based on 459 trees of a cross between the WBD-resistant ‘TSH 1188’ and the tolerant ‘CCN 51’ at the Mars Center for Cocoa Science (MCCS) in Barro Preto, Bahia, Brazil. Furthermore, we identified seven QTL on five different chromosomes that are associated with WBD using a multi-trait QTL analysis for outbreeders. Various potential candidate genes that were identified within the QTL may be involved in different steps in disease resistance. Potential SNP markers that can be applied in the marker-assisted selection of WBD-resistant material are proposed.

## Methods

### Plant material and design of the field experiment

For this study, a segregating population ‘MP01’ consisting of 459 offspring from a cross between ‘TSH 1188’ and ‘CCN 51’ was used. Both parents are very different for various traits of interest and the offspring segregates for pod color [6], self-compatibility, resistance to WBD, black pod and Ceratocystis wilt, fat content and fat composition, methylxanthines (theobromine and caffeine), flavanol content and some other agronomic traits. ‘TSH 1188’ is considered resistant to WBD, whereas ‘CCN 51’ is considered moderately resistant. A recent study focused on the number of cushion brooms showed that ‘TSH 1188’ had about 3 % infected flower cushions, whereas ‘CCN 51’ has close to 30 % infected flower cushions [20]. Individual trees were planted in 2000 in a 3 × 3 m grid in a hydromorphic and typical tropudalf (Itabuna modal) mixed soil type. Every fifth row in the field contained 5–19 trees (depending on the length of the rows) of the variety ‘Comum’, which is susceptible to WBD and serves as a natural and permanent inoculum source. The layout of the field experiment is shown in Additional file 1. Shade was provided using the traditional ‘cabruca’ system in which the trees are grown amongst the Atlantic Forest's native canopies.

### SNP identification, SNP array evaluation and genotyping of trees using the Illumina Infinium SNP chip

DNA for genotyping was isolated from leaf samples according to the protocol described by Motamayor et al. [6]. The SNPs that were used on the cacao Illumina Infinium SNP chip were identified as part of the work to identify conserved ortholog set II (COSII) SNP markers for MAS in cacao as described by Kuhn et al. [21] and Livingstone et al. [22]. The strategy was to sequence leaf RNA samples of 15 members of a panel of diverse cacao genotypes and to align the sequences against the *Theobroma cacao* Matina 1–6 leaf transcriptome. After alignment, a variant report was generated for all of the identified SNPs [21]. Further filtering of the SNPs was performed to select the final 6,000 SNPs to produce the 6 K SNP chip [22]. Of the 6,000 SNPs that were submitted for inclusion on the bead array, 5,388 were successfully synthesized. Failing to produce genotypic data for any sample, 174 SNPs were removed before analysis. Additionally, to serve as a quality control for genotype calling, only the markers with GenTrain and 10 % GenCall scores greater than 0.4 and 0.2, respectively, were included for data analysis. This process resulted in a final set of 5,149 SNPs. The Cacao6kSNP array was originally run with 1,152 DNA samples (including duplicated controls), but seven DNA samples failed completely. In total, 5,149 SNPs were genotyped in 1,145 DNA samples for a total of 5,895,605 data points or SNP genotypes [22].

### SNP genotype data analysis and construction of a genetic map

JoinMap®4.1 [23] was used to create the genetic map. SNPs with more than 5 % missing data were discarded. SNPs were also checked for strongly deviating segregation ratios. For each linkage group, the marker data were analyzed per segregation type (markers segregating only in the mother ‘TSH 1188’, markers segregating only in the father ‘CCN 51’, and cosegregating markers). Finally, integrated genetic maps were obtained using the Maximum Likelihood (ML) mapping algorithm [24]. Markers with a nearest-neighbor stress greater than 2 were discarded, followed by recalculation of the marker order. Map distances were calculated using the Haldane mapping function and the resulting maps were drawn using MapChart 2.1 [25].

### Phenotypic evaluation of WBD resistance

VB and cushion brooms (CB) were counted once or twice a year over a period of 4 years (April 2008, March 2009, March 2010, December 2010 and December 2011) and removed after they had been counted. The data from the 459 genotyped trees were recorded as the number of VB per year and the number of CB per year.

### QTL analysis

The distributions of the numbers of VB and CB per year were extremely skewed with many zeros. Therefore, the presence/absence of brooms*,* being the main feature of the phenotypic variation, was used for further analysis. To get an indication of the genetic component of variation for the number of VB and CB over time, determination coefficients were calculated from the variance components for genotypes and the genotype*-*by*-*year interaction using the REML facilities of GenStat 15 [26].

The number of years in which each tree carried VB and the number of years in which it carried CB were used for further analysis; the values for each of these traits varied between zero and four. To remove the effect of possible spatial patterns of infestation by WBD, the data was adjusted for row and column effects using the REML facilities of GenStat 15 [26]. The adjusted data of the two traits was used for a multi-trait QTL analysis [27], which was carried out using the facilities for QTL analysis in GenStat 15. The QTL profiles were obtained using all of the available markers using the integrated linkage map. The values of the test statistic (expressed as -log_10_(P)) are based on the additive effect of ‘TSH 1188’, the additive effect of ‘CCN 51’, and the interaction effect. The initial selection of QTL was performed by simple interval mapping using a threshold of three for the -log_10_(P), and the final selection of QTL was performed by backward elimination using the default parameters provided by GenStat.

To obtain a better sense of the precision of the QTL positions and their usefulness for cacao breeding, a simulation study was carried out in which random samples of 200 individuals were drawn from the original 459 individuals. For each sample, a QTL analysis was carried out as described above. The sample size of 200 was chosen as a reasonable size compared to the population size of many QTL experiments. For each sample, the marker positions that were selected as QTL were recorded as ‘hit’. The results of 500 random samples were stored, and a graphical display of the results was obtained using the procedure DQSQTLSCAN of GenStat 15.

### Identification of associations between haplotypes and WBD resistance

Haplotypes of the offspring and the parental haplotypes surrounding the SNP markers on each chromosome with a significant association with WBD resistance in the QTL analysis were identified using version 1.1 of the Matina 1–6 genome and iXora [6, 28]. To test the significance of the haplotype-phenotype associations, a chi-squared test was applied independently for each identified marker, based on the number of total VB as a trait, where the trees with more than 10 brooms were considered susceptible. ‘TSH 1188’ has two parental haplotypes that are designated as T1 and T2, whereas ‘CCN 51’ has the two parental haplotypes, C1 and C2. Chi-squared tests were also used to test whether the parental haplotype combinations that were present in the offspring (T1-C1, T1-C2, T2-C1 and T2-C2) were non-randomly associated with WBD resistance.

### Origin of disease resistance alleles

The phasing of 1162 individuals including three mapping populations and a set of diversity panel members [6] from distinct *T. cacao* structural groups was run with fastPHASE [29]. Sixty-two markers (from Tcm009s07051488 to Tcm009s09263083) in the WBD QTL9.1 region were used for the phasing. To improve the accuracy of phasing and to account for haplotype structure, a subpopulation label was used in the form of an integer. Known members that were sampled from the same population (i.e., mapping populations) were assigned the same population, while unrelated individuals were assigned a distinct integer. The expectation-maximization (EM) algorithm for computing the maximum likelihoods was controlled by the following options: 20 random starts, 25 iterations, and 200 haplotypes that were sampled from the posterior distribution from a particular random start. The default allelic two-parameter error model for inferring true genotypes was also used to scan for genotype errors. To create the phylogenetic neighbor joining (NJ) tree, thirty diversity panel members were chosen, including the parents from MP01, CATIE type 1, and PNG mapping populations [6]. The distance matrix for phylogeny estimation was created with the MEGA 6 phylogeny reconstruction setting, using the method of Maximum Composite Likelihood using 1000 bootstrap samples [30].

### Candidate gene identification

Using the location of the identified SNP markers with the highest association in the QTL analysis and the gene annotations in the version 1.1 Matina 1–6 genome [6], a search was performed manually for possible candidate genes involved in disease resistance. The specific keywords that were searched for were resistance, senescence, jasmonic acid, salicylic acid (SA), ethylene (ET), gibberellic acid, auxin, reactive oxygen species (ROS), mitogen-activated protein kinases (MAPKs), F-box, LRR-receptor, cysteine, serine/threonine protein kinase, phosphatase, ubiquitin, NPR1, and WRKY. All of the genes within an interval of approximately 2 cM surrounding the most significant marker for each QTL were investigated in more detail using BLASTX and additional literature study. All details about the experimental set-up and data analysis for the differential gene expression of a subset of candidate genes are given in the Additional file 2 and in Additional file 3.

## Results and discussion

### Construction of the integrated genetic map and relationship with the physical map

**Fig. 1:**
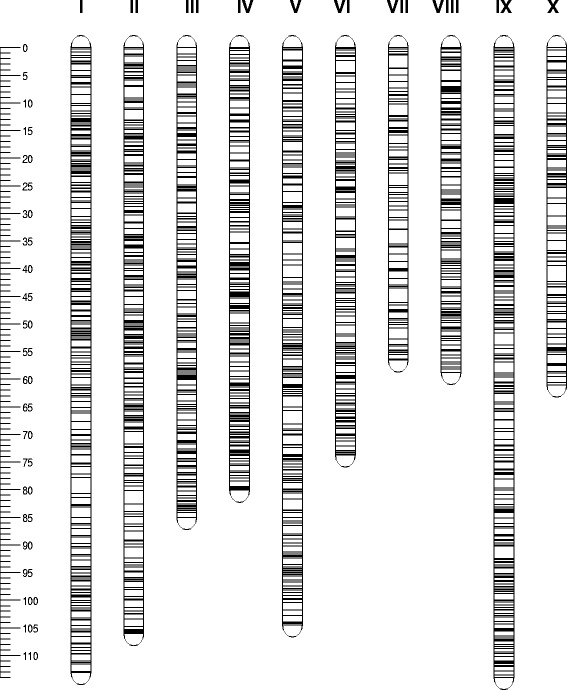
Integrated linkage map of MP01. The integrated linkage map is based on a population of 459 individuals obtained from a cross between ‘TSH 188’ and ‘CCN 51’. The linkage map contains 3,526 SNPs with segregation types ab × aa (1,192 SNPs), aa × ab (1,100 SNPs) and ab × ab (1,251 SNPs)

For the construction of the MP01 map, 3,564 segregating SNPs could be scored: 1,198 SNPs were classified as segregating in ‘TSH 1188’ (segregation type: ‘ab × aa’), 1,105 SNPs as segregating in ‘CCN 51’ (‘aa × ab’) and 1,261 as segregating in both parents (‘ab × ab’). Eleven SNPs were discarded because they had more than 5 % missing data. Nine SNPs that were classified as segregating in both parents were discarded because for these markers, the offspring individuals were scored in only two classes instead of three, leaving 3,544 SNPs for further analysis. Using a recombination frequency threshold of 0.2, ten major linkage groups were formed. One marker from chromosome III (according to its location on the assembled genome) was discarded because it was unlinked from other markers of the same segregation type. Additionally, 17 markers were removed after map inspection because of Nearest Neighbor Stress (N.N. Stress) values greater than 2.0 cM (three markers on chromosome III, three markers on chromosome V, and 11 markers on chromosome IX). An overview of the map construction process is given in Table 1. As an example, Additional file 4 shows the agreement between the linkage maps for chromosome IX that were obtained with markers of the segregation types ‘ab × aa’, ‘ab × ab’ and ‘aa × ab’ and the integrated map of chromosome IX. The integrated linkage maps for every chromosome are shown in Fig. 1.

**Table 1:** An overview of the mapping process

Chromosome	Number of SNPs				Map length (cM)			
	ab × aa	aa × ab	ab × ab	All	ab × aa	aa × ab	ab × ab	All
I	153	114	209	476	98.7	109.2	106.6	113.1
II	138	141	183	462	100.2	105.5	99.9	106.0
III	95	155	115	362^a^ (365)	78.1	80.6	84.4	85.0
IV	163	122	145	430	70.1	72.7	71.5	80.1
V	150	121	122	390^a^ (393)	88.7	90.8	86.6	104.5
VI	70	107	106	283	58.1	70.2	67.6	73.7
VII	37	46	73	156	51.1	64.2	54.9	56.5
VIII	85	91	103	279	53.0	53.8	53.9	58.8
IX	234	151	148	522^a^ (533)	100.9	107.2	104.2	114.0
X	67	52	47	166	53.9	59.9	54.6	61.1
Total	1,192	1,100	1,251	3,526^a^ (3,543)	752.8	814.1	784.2	852.8

^a^Some markers were removed from the final map because of Nearest Neighbour Stress (N.N. Stress) values greater than 2.0, of which three markers on chromosome III, three markers on chromosome V, and 11 markers on chromosome IX

Compared to the most recent map [19], the MP01 map contains three times more markers (3,526 vs. 1,043), has a map length that is 12 % longer (852.8 cM vs. 751.7 cM) and has a marker density that is three times denser (0.24 cM vs. 0.72 cM). The shortest chromosome was chromosome VII (56.5 cM and 156 SNPs), and the longest chromosome was chromosome IX (114.0 cM and 522 SNPs). The average gap without SNP markers was 2.3 cM, with the smallest gap of 1.8 cM on chromosomes III and VIII and the largest gap of 3.0 cM on chromosome X. The average distance between two markers varied from 0.19 cM on chromosome IV to 0.37 cM on chromosome X (Table 1**,** Additional file 5). In contrast to the previously published studies [15–19, 31], no distorted segregation ratios were observed; all SNPs of the type ‘ab × aa’ and ‘aa × ab’ showed a segregation ratio between 0.4 and 0.6 (Additional file 6). The relationship between the positions of the SNPs on the integrated linkage map and the corresponding position on the physical map is shown in Additional file 7. Apart from a few deviating points, a nearly perfect correspondence exists between the linkage map and the physical map. All ten chromosomes show physical regions with reduced recombination rates, presumably the centromeric regions, which agrees with the karyotyping results and demonstrates the lack of a long arm for some of the chromosomes and that chromosomes VI, VII, VIII and X are shorter than the other chromosomes [6].

### Phenotypic evaluation of WBD resistance

‘TSH 1188’ is considered resistant to WBD, whereas ‘CCN 51’ is considered moderately resistant. A recent study in MP01 focused on the number of cushion brooms showed that ‘TSH 1188’ had about 3 % infected flower cushions, whereas ‘CCN 51’ had close to 30 % infected flower cushions [20]. Nowadays we notice that ‘CCN 51’ has many more infected flower cushions located on the stem and the lower part of the main branches, compared to ‘TSH 1188’, but the latter one seems to have more infected flower cushions higher up in the canopy. Nevertheless, the number of vegetative brooms is much lower in ‘TSH 1188 than in ‘CCN 51’. It is also noteworthy that both clones are as good as resistant for WB infection of the pods. There also exist region-specific differences, which are most likely associated with differences in existing pathogen strains. It is therefore important to mention that the outcome of a plant-pathogen interaction does not depend solely on the plant genotype and it may change completely depending on the strain of the pathogen.

For each genotype, the total numbers of VB and CB per year were used (Additional file 8). For both traits, the distributions were highly skewed and contained many zeros. The average number of VB per year ranged from 2.1 to 4.0, and the average number of CB per year ranged from 0.8 to 7.3. The maximum number of VB per year ranged from 26 to 56, and the maximum number of CB per year ranged from 24 to 139 (Additional file 9a). Over time, the percentage of trees actually carrying brooms varied between 51.85 and 64.05 for VB and between 20.26 and 64.71 for CB (Table 2). Therefore, we focused on the presence or absence of brooms rather than on their actual numbers (Additional file 9b). The coefficients of determination based on the presence/absence were 0.55 for VB and 0.53 for CB, indicating that slightly more than half of the variation in genotypic means (based on the averages over 4 years) can be attributed to differences between the genotypes. It should be noted here that genotypes are confounded with positions in the field, so that the genetic part of the coefficient of determination may be considerably lower than the values found. Due to possible spatial patterns of infestation, the data were adjusted for row and column effects, and the adjusted data were used for the QTL analysis (Additional file 9c).

**Table 2:** Proportions of trees in MP01 with vegetative brooms and cushion brooms in the years 2008–2011

Year	Vegetative brooms	Cushion brooms
2008	51.85	64.71
2009	64.05	20.26
2010	54.47	37.04
2011	63.40	54.25
Average	58.44	44.07

### Identification of QTL for WBD resistance

**Fig. 2:**
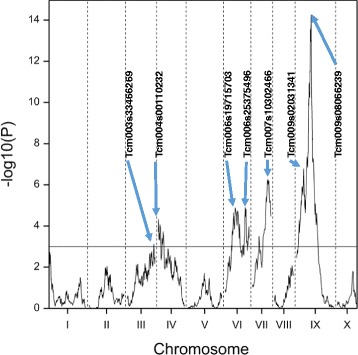
Graphical results of the multi-trait QTL analysis. The multi-trait QTL analysis on VB and CB, adjusted for row and column effects in the experimental set-up. The markers associated with the QTL after backward selection are pointing towards the selected peaks

The multi-trait QTL analysis identified various QTL, but after backward selection on VB and CB (adjusted for row and column effects in the experimental set-up) seven positions on five different chromosomes were retained as potential QTL (Fig. 2**)**. All seven positions exhibited QTL effects that differ in the two traits under study. A summary of the results after backward elimination is shown in Table 3.

**Table 3:** Effects of selected QTL, values of the significance level -log_10_(*P*) and % variance accounted for by including markers in the final QTL model

Marker	Chromosome	Position (cM)	-log_10_(*P*)	Trait	TSH-1188	CCN-51	TSH 1188 × CCN 51	% variance
**Tcm003s33466269**	III	81.53	3.739	VB	0.069 (0.0483)	0.086 (0.0480)	−0.025 (0.0479)	0.6
				CB	0.054 (0.0449)	**0.209 (0.0446)**	0.032 (0.0445)	3.3
**Tcm004s00110232**	IV	0.55	6.031	VB	**0.177 (0.0483)**	**−0.111 (0.0487)**	0.015 (0.0486)	2.9
				CB	**0.248 (0.0455)**	−0.033 (0.0452)	0.014 (0.0452)	4.7
**Tcm006s19715703**	VI	31.01	3.679	VB	**−0.107 (0.0520)**	**0.190 (0.0551)**	**0.118 (0.0496)**	5.2
				CB	−0.059 (0.0484)	**0.120 (0.0512)**	0.036 (0.0461)	2.5
**Tcm006s25375496**	VI	61.73	2.318	VB	0.015 (0.0551)	0.015 (0.0551)	−0.097 (0.0491)	3.1
				CB	**−0.169 (0.0483)**	0.0206 (0.0512)	**−0.111 (0.0456)**	5.4
**Tcm007s10302466**	VII	47.55	8.306	VB	**−0.105 (0.0478)**	**−0.105 (0.0478)**	−0.080 (0.0486)	6.3
				CB	**0.146 (0.0452)**	**−0.100 (0.0444)**	**−0.139 (0.0452)**	3.4
**Tcm009s02031341**	IX	13.73	3.557	VB	**−0.221 (0.0579)**	**−0.221 (0.0579)**	−0.094 (0.0500)	6.0
				CB	−0.065 (0.0498)	**−0.203 (0.0538)**	−0.024 (0.0465)	1.7
**Tcm009s08066239**	IX	44.36	11.24	VB	**0.383 (0.0539)**	0.057 (0.0573)	−0.062 (0.0503)	13.5
				CB	**0.239 (0.0501)**	0.110 (0.0533)	−0.049 (0.0467)	6.2

The additive effect of ‘TSH 1188’ ( or ‘CCN-51’) represents the average difference in response between individuals with the allele of the first grandparent ‘TSH-1188’ (or ‘CCN-51’) and the allele of the second grandparent of ‘TSH 1188’ (or ‘CCN 51’). The interaction ‘TSH-1188’ × ‘CCN-51’ represents the average difference in response between individuals with the allele of the first grandparent of ‘TSH-1188’ and the allele of the second grandparent ‘TSH-1188’ in combination with the allele of the first grandparent of ‘CCN 51’ and the same average difference in combination with the allele of the second grandparent of ‘CCN 51’. The figures between brackets represent standard errors. The designation as first and second grandparent depends on the phasing of the marker alleles and determined during the construction of the linkage map. The values in bold are values that are at least two times larger than their standard error

Trait refers to the number of years in which vegetative brooms (VB)/cushion brooms (CB) occurred, adjusted for spatial effects

The major QTL (for both VB and CB) on chromosome IX most likely corresponds to the QTL identified in the F_2_ population obtained by selfing an individual of a cross between the WBD-resistant ‘SCA 6’ and the WBD-susceptible ‘ICS 1’ [12–14]. This result might not be surprising because ‘SCA 6’ is present in the parental lineage of ‘TSH 1188’. ‘TSH 1188’ results from a cross between ‘POUND 18’ and ‘TSH 753’ [32, 33]; ‘TSH 753’ is the result of an open-pollination of ‘TSA 641’ [33, 34], and ‘TSA 641’ is the result of a cross between ‘SCA 6’ and ‘IMC 67’ [33, 35]. The second QTL on chromosome IX had an additive effect in both ‘TSH 1188’ and ‘CCN 51’ for VB, but had an effect only in ‘CCN 51’ for CB. Other ‘CCN 51’ specific effects for CB were identified for the QTL on chromosome III and on chromosome IV. Similar effects in ‘CCN 51’ for various other QTL were noticed, indicating that these newly discovered QTL might contribute to the known moderate resistance/tolerance of ‘CCN 51’ to WBD and can be used as alternative sources of resistance. ‘CCN 51’ is derived from crosses involving ‘IMC 67’ and ‘ICS 95’ [36]. ‘IMC 67’ shows resistance or susceptibility to WBD according to different studies [33, 37, 38], whereas ‘ICS 95’ has intermediate resistance [33, 39, 40]. At MCCS, the two clones show an intermediate level of resistance to WBD. The fact that about 10–17 % of the trees in the offspring doesn’t have any CB or VB (Additional file 8), suggests that they may have a combination of favorable alleles originating from ‘SCA 6’, ‘IMC 67’ and ‘ICS 95’. The higher number of resistant trees (about 65 %) in MP01 might indicate that they have a combination of favorable alleles from any of these three clones. A preliminary study performed by Santos et al. [41] used 18 SSRs (some linked to resistance in the F_2_ mapping population [12–14]) on 16 trees (eight resistant and eight susceptible) of a subset of 50 trees of MP01 under natural and artificial infection pressure with *M. perniciosa*. A simple regression analysis indicated that in addition to the known markers on chromosome IX, there were also markers that showed an association with resistance on chromosomes I, III and IV. The markers on chromosomes III and IX were from the ‘TSH 1188’ parent, whereas the markers on chromosomes I and IV were from the ‘CCN 51’ parent, most likely indicating an additional source of resistance [41]. In the present study, however, no QTL were located at chromosome I. The identified QTL on chromosomes III and IV were located on the opposite side of the chromosomes compared to our results. Alternative QTL were identified in ‘CAB 208’ and ‘CAB 214’, which were originally collected from the Brazilian Amazon and did not correspond to the QTL that were identified in this study [11]. These results suggest that there are multiple sources of resistance in the different mapping populations for both VB and CB. To better understand the precision of the QTL positions and their usefulness for cacao breeding, a resampling study was carried out in which random samples of 200 individuals were drawn 500 times from the original 459 individuals, and the results are shown in Additional file 10. The sample size of 200 individuals was chosen because most of the published QTL results in cacao are based on a population size ranging from 95 to 264 individuals [12, 15–19, 31, 42–44]. For the region between 40.8 cM and 45.1 cM of chromosome IX, the average percentage of samples in which a marker position is selected as a QTL (= %hit) was 84.0 %; nearly half of these samples (41.2 %) were at 44.4 cM. For a slightly larger region between 37.9 cM and 47.5 cM, the %hit was 94.0 %. For the region between 5.2 and 18.3 cM of chromosome IX, the %hit was 15.8 %. The hits on the other chromosomes were more dispersed (Additional file 10). From the simulation study, it became clear, however, that the more trees are used in the study, the more precise the QTL analysis becomes, smaller QTL effects can detected, but also the positions of the QTL with small effects are not very accurate.

### Identification of associations between the parental haplotypes and WBD resistance

**Fig. 3:**
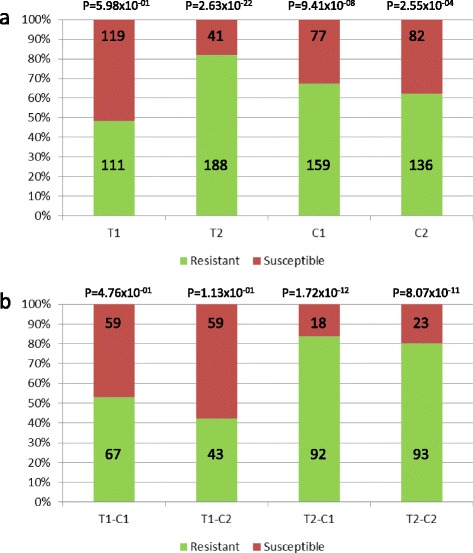
Distribution of resistant and susceptible trees. The distribution of resistant and susceptible trees is given for (**a**) each parental haplotype for QTL9.1, and (**b**) each parental haplotype combination for QTL9.1. The x-axis represents the different haplotypes and haplotype combinations, respectively, whereas the y-axis represents a stacked column representing the proportion of resistant vs. susceptible trees (*in percent*). From (**a**) it is clear that the T2 haplotype has more resistant trees (i.e. 82 %) than the other three haplotypes. From (**b**) considering the parental haplotype combinations, the combination T2-C1 has most of the resistant trees (84 %), followed by T2-C2 (80 %). The numbers in the boxes indicate the number of trees in each class, and the p-values show whether they are significantly different from each other

The parental haplotypes of the markers that were associated with the QTL are presented in Table 4. An analysis of the parental haplotype-phenotype associations based on the number of resistant/susceptible trees (the threshold for susceptible trees was set for trees with more than ten total VB) for single QTL showed that the QTL9.1_T2 haplotype (SNP marker Tcm009s08066239, G-allele) had the highest association of all QTLs with a P-value of 2.63 × 10^−22^ (Table 5). Of the 299 resistant trees in the mapping population of 459 trees, 188 trees (62.9 %) possessed the T2 haplotype at this marker; however, 41 trees that carry the favorable T2 haplotype were susceptible to WBD (data not shown). For the haplotype combinations in the offspring (indicated as T1C1, T1C2, T2C1 and T2C2 with *T* = ‘TSH 1188’ and *C* = ‘CCN 51’), the highest associations were found for QTL9.1_T2-C1 (chi-square test, *P* = 1.72 × 10^−12^) (Table 6). The frequency distributions of resistant and susceptible trees for each haplotype and the parental haplotype combinations for QTL9.1, presented in Fig. 3, show that approximately 82 % of the trees with the T2 haplotype for this marker were resistant to WBD. When considering the different parental allele combinations, QTL9.1_T2-C1 showed the highest association but was only slightly higher than QTL9.1_T2-C2 with approximately 84 and 80 % resistant trees, respectively.

**Table 4:** Alleles for each of the parental haplotypes (T1, T2, C1 and C2) of ‘TSH 1188’ and ‘CCN 51’ for each QTL and representative SNP marker associated with WBD

QTL	SNP marker	TSH 1188		CCN 51	
		Allele T1	Allele T2	Allele C1	Allele C2
QTL3.1	Tcm003s33466269	G	G	G	**T**
QTL4.1	Tcm004s00110232	**T**	C	C	C
QTL6.1	Tcm006s19715703	T	T	T	**C**
QTL6.2	Tcm006s25375496	G	**T**	G	G
QTL7.1	Tcm007s10302466	G	G	**A**	G
QTL9.1	Tcm009s08066239	A	**G**	A	A
QTL9.2	Tcm009s02031341	A	**G**	A	A

The alleles marked in bold are segregating in MP01 and are also the favorable alleles associated with WBD resistance

**Table 5:** Chi-squared test to calculate the associations between the parental haplotypes (T1, T2, C1 and C2) and the observed and expected number of resistant/susceptible trees

QTL and haplotypes	Total	Observed		Expected		P-value	% resistant
		Resistant	Suscept.	Resistant	Suscept.		
**QTL3.1_T1**	242	154	88	121	121	2.21E-05	63.64
**QTL3.1_T2**	217	145	72	108.5	108.5	7.21E-07	66.82
**QTL3.1_C1**	211	147	64	105.5	105.5	1.10E-08	69.67
**QTL3.1_C2**	248	152	96	124	124	3.77E-04	61.29
**QTL4.1_T1**	244	144	100	122	122	4.85E-03	59.02
**QTL4.1_T2**	215	155	60	107.5	107.5	9.24E-11	72.09
**QTL4.1_C1**	259	167	92	129.5	129.5	3.16E-06	64.48
**QTL4.1_C2**	200	132	68	100	100	6.03E-06	66.00
**QTL6.1_T1**	221^a^	127	94	110.5	110.5	2.64E-02	57.47
**QTL6.1_T2**	232^a^	167	65	116	116	2.13E-11	71.98
**QTL6.1_C1**	228	135	93	114	114	5.41E-03	59.21
**QTL6.1_C2**	231	164	67	115.5	115.5	1.75E-10	71.00
**QTL6.2_T1**	221	125	96	110.5	110.5	5.11E-02	56.56
**QTL6.2_T2**	238	174	64	119	119	1.00E-12	73.11
**QTL6.2_C1**	216^a^	132	84	108	108	1.09E-03	61.11
**QTL6.2_C2**	239^a^	165	74	119.5	119.5	3.95E-09	69.04
**QTL7.1_T1**	234	174	60	117	117	9.16E-14	74.36
**QTL7.1_T2**	225	125	100	112.5	112.5	9.56E-02	55.56
**QTL7.1_C1**	230	138	92	115	115	2.42E-03	60.00
**QTL7.1_C2**	229	161	68	114.5	114.5	7.97E-10	70.31
**QTL9.1_T1**	230	111	119	115	115	5.98E-01	48.26
**QTL9.1_T2**	229	188	41	114.5	114.5	**2.63E-22**	82.10
**QTL9.1_C1**	236^a^	159	77	118	118	9.41E-08	67.37
**QTL9.1_C2**	218^a^	136	82	109	109	2.55E-04	62.39
**QTL9.2_T1**	224	127	97	112	112	4.50E-02	56.70
**QTL9.2_T2**	235	172	63	117.5	117.5	1.16E-12	73.19
**QTL9.2_C1**	219	159	60	109.5	109.5	2.23E-11	72.60
**QTL9.2_C2**	240	140	100	120	120	9.82E-03	58.33

The threshold for susceptible trees was set for trees with more than ten total vegetative brooms over the whole period of 4 years

‘Total’ indicates the total number of trees in MP01 that have that particular haplotype for the specific QTL. ‘^a^’ indicates that certain haplotypes could not be exactly identified due to recombination at that particular locus, and the resulting haplotype could be one of both parental haplotypes

P-value in bold indicates the highest association identified between the various QTL/haplotype combinations and WBD resistance

**Table 6:** Chi-squared test to calculate the associations between the parental haplotype combinations (T1-C1, T1-C2, T2-C1 and T2-C2) and the number of resistant/susceptible trees

QTL 1	QTL 2	QTL 3	Total	Observed		Expected		P-value	% resistant
				Resistant	Suscept.	Resistant	Suscept.		
QTL9.1_T2-C1	NA	NA	110	92	18	55	55	1.72E-12	83.6
QTL6.1_T2-C2	NA	NA	128	101	27	64	64	6.12E-11	78.9
QTL9.1_T2-C2	NA	NA	116	93	23	58	58	8.07E-11	80.2
QTL7.1_T1-C2	NA	NA	111	88	23	55.5	55.5	6.85E-10	79.3
QTL9.2_T2-C1	NA	NA	117	90	27	58.5	58.5	5.73E-09	76.9
QTL9.1_T2-C1	QTL9.2_T2-C1	NA	68	59	9	34	34	1.33E-09	86.8
QTL9.1_T2-C2	QTL6.1_T2-C2	NA	37	35	2	18.5	18.5	5.79E-08	94.6
QTL9.1_T2-C2	QTL6.2_T2-C2	NA	41	37	4	20.5	20.5	2.55E-07	90.2
QTL9.1_T2-C1	QTL7.1_T1-C1	NA	34	32	2	17	17	2.68E-07	94.1
QTL9.1_T2-C2	QTL9.2_T2-C2	NA	71	57	14	35.5	35.5	3.34E-07	80.3
QTL9.1_T2-C2	QTL6.1_T2-C2	QTL6.2_T2-C2	20	19	1	10	10	0.000057	95.0
QTL9.1_T2-C2	QTL9.2_T2-C2	QTL6.1_T2-C2	23	21	2	11.5	11.5	0.000074	91.3
QTL9.1_T2-C1	QTL9.2_T2-C1	QTL3.1_T2-C1	18	17	1	9	9	0.000162	94.4
QTL9.1_T2-C2	QTL9.2_T2-C2	QTL7.1_T1-C2	18	17	1	9	9	0.000162	94.4
QTL9.1_T2-C2	QTL9.2_T2-C2	QTL6.2_T2-C2	24	21	3	12	12	0.000239	87.5

The threshold for susceptible trees was set for trees with more than ten total vegetative brooms over the whole period of 4 years. For the combinations of two or three different QTLs, the first QTL was always QTL9.1 since it showed the highest association as a single QTL

‘NA’, not applicable

To identify the best marker combinations of two QTL for the selection of resistant trees, each haplotype combination of QTL9.1 was combined with each haplotype combination for the other QTL. All the associated P-values are presented in Additional file 11, but the five highest associations for a combination of two QTL are presented in Table 6. The highest association was found for QTL9.1_T2-C1 with QTL9.2_T2-C1 (*P* = 1.33 × 10^−9^), followed by QTL9.1_T2-C2 with QTL6.1_T2-C2 (*P* = 5.79 × 10^−8^).

To identify the best marker combinations of three QTL for the selection of resistant trees, two haplotype combinations of QTL9.1 (T2-C1 and T2-C2) were combined with the more-significant haplotype combinations for two other QTL. All of the associated P-values are presented in Additional file 11, but the five highest associations for a combination of three QTL are presented in Table 6. The highest association was found for the combination of QTL9.1_T2-C2 with QTL6.1_T2-C2 and QTL6.2_T2-C2 (*P* = 0.000057), followed by the combination of QTL9.1_T2-C2 with QTL9.2_T2-C2 and QTL6.1_T2-C2 (*P* = 0.000074). The percentage of resistant trees for the top five of each QTL combination varied between 76.9 and 95.0 % depending on the number of trees that were available with that specific combination (Table 6).

Furthermore, we used the trees that showed a recombination event between the different haplotypes within a small region around the most significant marker and extended this region by adding flanking SNP markers. This extension was designed to help us to localize more precisely the most likely candidate genes, but we only managed to do this successfully for the most significant QTL9.1 on chromosome IX (Additional file 12). Between the markers Tcm009s08010009 and Tcm009s08051015, we identified three trees (MP01-212, 555 and 795) with a recombination event between the maternal haplotypes. Between Tcm009s08051015 and the most significant marker Tcm009s08066239, we identified a single tree (MP01-218) with a recombination event between the maternal haplotypes. One recombination event (MP01-462) was observed between Tcm009s08066239 and Tcm009s08105371. Furthermore, we identified two recombination events (MP01-243 and 593) between the markers Tcm009s08105371 and Tcm009s08172795 (Additional file 12). Based on the recombinants, the size of QTL9.1 between the first and the last SNP marker is 162,786 bp and contains five candidate genes possibly involved in disease resistance (see below).

For the most significant marker (Tcm009s08066239) and its neighbor (Tcm009s08051015), all of the trees that have the T2 haplotype, i.e., the G allele or the T allele, respectively, had ten or fewer total VB over the 4 years and can be considered resistant (Additional file 12). The resistant trees that carry the A allele (in orange, Additional file 12) possess one or more associated alleles of the other identified QTL (data not shown). The susceptible trees (more than ten total VB, in red, Additional file 12), with the exception of MP01-114, do not possess any of the associated alleles of the other identified QTL. MP01-114 contains the favorable alleles of QTL6.1_T2-C2, QTL6.2_T2-C2 and QTL4.1_T2-C1. Sixty-four trees with a single recombination event were identified in a larger interval from 7,293,462 to 9,668,548 bp. Of the 18 susceptible trees within this interval, only MP01-281 has the G allele, i.e., only 6 % of the trees carrying the G allele are at risk of being misidentified as being resistant based on the allele information (data not shown).

This result also indicates that these allele-specific markers, either by themselves or in combination with some of the other allele-specific markers of the other QTL, can be used for the diagnostic disease resistance screening of other populations and germplasm collections. The associated SNP markers can also be relatively easily converted into PCR-based markers using the dCAPS method [45].

### Origin of disease resistance alleles

**Fig. 4:**
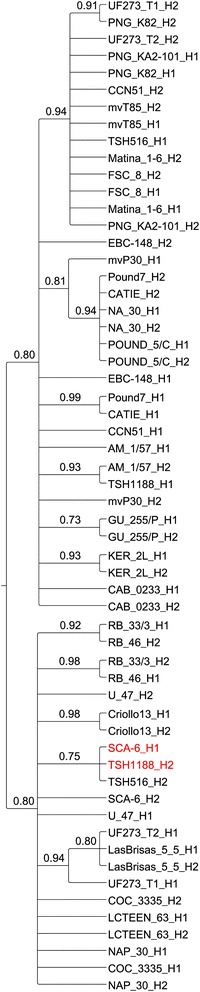
Neighbor Joining tree identifies the origin of the main QTL9.1 to ‘SCA 6’. Sixty-two markers in the QTL9.1, spanning about 500 kb, were used for the phasing, which permitted generation of haplotype sequences and, consequently, identification of the alleles associated with WBD in the QTL9.1

Sixty-two markers in the WBD QTL9.1, spanning approximately 500 kb, were used for the phasing, which permitted the generation of haplotype sequences and the identification of the alleles that were associated with WBD. The second haplotype of ‘TSH 1188’, corresponding to the G allele and conferring resistance to WBD, grouped with ‘SCA 6 H1’ (Fig. 4). This result is not surprising because ‘SCA 6’ is one of the great grandparents of ‘TSH 1188’, together with ‘IMC 67’ [32–35], and is a major source of WBD resistance [10]. This haplotype also groups with ‘TSH 516 H2’, which is the result of another cross of ‘SCA 6’ with ‘ICS 1’, the latter being susceptible to WBD [33, 35]. The first haplotype of ‘TSH 1188’, corresponding to the A allele, grouped closely with the first haplotype of ‘CCN 51’ and with both haplotypes of ‘AM 1/57’. The second haplotype of ‘CCN 51’ grouped with ‘TSH 516’ H1 and both of the Matina 1–6 haplotypes (Fig. 4).

### Potential candidate genes possibly involved in Witches’ broom resistance through programmed cell death

All of the genes within an interval of 2–3 cM (approximately 300,000 to 700,000 bp) surrounding the most significant marker for each QTL were investigated. The number of genes within each QTL ranged from 23 in QTL9.1 to 90 in QTL6.1, and the number of candidate genes that were potentially involved in disease resistance ranged from 6 in QTL9.1 to 29 in QTL6.1 (Additional file 13). The potential biological processes for each of the genes were determined by GO Slim annotation (Additional file 14).

One of the first steps in the recognition of pathogens is the activation of plant nucleotide-binding (NB)-leucine-rich repeat (LRR) receptors by direct or indirect mechanisms [46]. On QTL6.1, three putative disease resistance-related proteins were identified that contain the NB and LRR domains. Thecc1EG028968 and Thecc1EG028973 encode a CC-NBS-LRR resistance protein, whereas Thecc1EG028972 encodes an NB-ARC domain-containing disease resistance protein. A fourth gene, Thecc1EG028979 encodes a disease resistance-responsive family protein. The SNP Tcm006s19715703, which is associated with QTL6.1, is located within Thecc1EG028962, a gene encoding a hydroxyproline-rich glycoprotein family protein (HRGP). Cross-linking of HRGPs is an important process by which to strengthen the cell walls, contributing to plant defense reactions [47]. Another gene, Thecc1EG028959, in QTL6.1 is the leaf senescence-associated receptor-like protein kinase, which has homology to the senescence-induced receptor-like serine/threonine protein kinase (SIRK). This gene is specifically expressed in the leaves at senescence and after pathogen infection and is a putative target of WRKY6 [48], which is a transcription factor that is activated through the MAPK pathway [49]. On QTL6.2, the most associated SNP, Tcm006s25375496, is within Thecc1EG030009, a MYB-like 102 gene that does not seem to function in disease resistance.

The next stages of the plant immune response include a rapid influx of calcium ions, an oxidative burst of ROS and the activation of MAPKs [50–52]. Four genes that were identified in QTL7.1 have been annotated as 2-oxoglutarate and Fe(II)-dependent oxygenase superfamily proteins (Thecc1EG032367, Thecc1EG032371, Thecc1EG032376 and Thecc1EG032382), which are involved in the formation of hydrogen peroxide at different stages in cacao depending on the disease status of the trees (resistant vs. susceptible) [53, 54]. On QTL7.1, the most associated SNP, Tcm007s10302466, is within Thecc1EG032345, a nucleotide/sugar transporter protein, which does not seem to function in disease resistance. Another neighboring gene, Thecc1EG032344, is annotated as an aldolase-type TIM barrel family protein, which in *Arabidopsis thaliana* seems to be involved in defense response to bacteria and in the hydrogen peroxide biosynthetic process [55].

The MAPK signaling cascade is involved in the biosynthesis and signaling of plant stress hormones, ROS generation, stomatal closure, defense gene activation, phytoalexin biosynthesis, cell wall strengthening and programmed cell death (PCD) [51]. In *A. thaliana*, one of the genes involved in this cascade is MAPK kinase 5, which is also one of the genes (Thecc1EG016564) that we identified in QTL3.1 [46, 48, 51]. Another disease resistance-related gene, Thecc1EG016546, in QTL3.1 encodes a glycosyl hydrolase superfamily protein. In crucifers, these enzymes break down glucosinolates and convert them into biologically active compounds. These compounds restrain insect feeding activity on plants and have potent antimicrobial activity, mainly against necrotrophic fungi [56, 57]. NDR1 (Thecc1EG016558), which is in the same QTL, was shown to be required for disease resistance in *A. thaliana* to both bacterial and fungal pathogens [58]. The SNP marker Tcm003s33466269, which is associated with QTL3.1, is located within Thecc1EG016568, a gene encoding an RNA-binding (RRM/RBD/RNP motifs) family protein.

Another important pathway in disease response and PCD is the ubiquitin/26S proteasome-mediated pathway, which is involved in the selective degradation of proteins in cells of eukaryotic organisms [59–61]. At least five U-box E3 ligases have been identified that are involved in disease response to various pathogens in different plants species [62–67]. For example, disruption of the *A. thaliana* Plant U-box protein 13 (PUB13) by T-DNA insertion results in spontaneous cell death, the accumulation of hydrogen peroxide and SA, and elevated resistance to biotrophic pathogens, as well as increased susceptibility to necrotrophic pathogens [65]. Tcm009s08066239, the SNP in QTL9.1 with the best association with WBD, is located into Thecc1EG038262 that has been annotated as a RING/U-box superfamily protein. Another RING/U-box superfamily protein (Thecc1EG038259) has been identified within the same QTL. Three other potential candidate genes in this QTL have been annotated as follows: DNA-binding protein phosphatase 1 (Thecc1EG038249, dephosphorylation and inactivation of MAPK [51]), uveal autoantigen with coiled-coil domains and ankyrin repeats (UACA, Thecc1EG038261), and serine/threonine protein kinase CTR1/EDR1 with an octicosapeptide/Phox/Bem1p domain (Thecc1EG038267). In humans, UACA is involved in a series of molecular signals in which an intracellular signal is conveyed to trigger apoptotic cell death [68]. This pathway is induced by the detection of DNA damage, which can be caused by ROS, thereby linking to one of the primary symptoms of WBD. CTR1 is a Raf-like MAPKKK that is involved in ET signal transduction [69, 70], and EDR1 was found to encode a MAPKKK that is similar to CTR1. This MAPKKK is involved in the induction of several defense responses, including host cell death, and it negatively regulates SA-dependent defense responses, abscisic acid (ABA) signaling, and ET-induced senescence [71]. Tcm009s02031341, the SNP in QTL9.2 with the best association with WBD, is located within Thecc1EG047089 that has been annotated as encoding an RNA recognition motif (RRM)-containing protein. It is interesting that, according to STRING v9.1 [72], this protein in *A. thaliana* may interact with a TIR-NBS-LRR class disease resistance protein. Among the other candidate genes, two were annotated as BURP domain-containing proteins. These proteins are involved in abiotic and biotic stresses in Brassica [73].

Ca^2+^ and MAPKs also control the synthesis and/or signaling of the hormones that are involved in the later steps of defense gene activation and PCD. The SNP marker Tcm004s00110232 that is associated with QTL4.1 is located within Thecc1EG016777 encoding a UDP-glucose pyrophosphorylase 2. Overexpression of this enzyme in poplar showed extremely high quantities of the glycoside of salicylic acid (SAG). SA accumulation, and therefore SAG, could catalyze the increase in other plant defense metabolites [74]. Also in QTL4.1 Thecc1EG016815, the senescence-associated gene 101, is part of the Enhanced Disease Susceptibility 1 (EDS1) pathway and is responsible for SA accumulation after infection with biotrophic pathogens of *A. thaliana* [75].

Of the 11 candidate genes selected for differential gene expression (Additional file 3), only six showed considerable expression differences between infected and mock-infected plants for both ‘CCN 51’ and ‘TSH 1188’. The most remarkable expression differences were seen for the UACA gene. When comparing the infected ‘CCN 51’ plants, it was clear that this gene was approximately 4-fold upregulated at 3 and 12 h after infection (HAI), whereas it had a basal level of expression at the other time points. In the resistant ‘TSH 1188’, this gene was approximately 8-fold downregulated at 3 HAI, whereas it had a similar expression profile for the other time points (see Additional files 15 and 16). According to the study of Sena et al. [76] this early response may correspond with the early infection stage of the fungus, where the basidiospores start to germinate around 2 HAI in the susceptible ‘CATONGO’, and around 4 HAI in the resistant ‘TSH 1188’, but both reach their maximum at 6 HAI. Penetration into the plant tissues was observed at 6 HAI for both susceptible and resistant genotypes, and in susceptible genotypes, primary hyphae were observed in the cortex under the epidermis at 48 HAI [76].

A study performed by Teixeira et al. [77] showed a detailed transcriptome analysis of the interaction between *M. perniciosa* and cacao at 30 days after artificial infection (which corresponds with the green broom stage). They identified 1,269 upregulated and 698 downregulated differentially expressed genes in cacao. Of those differentially expressed genes 26 were located within our identified QTL. Three of those genes were pathogen-related (CDG0008741 in QTL6.2) or receptor-like kinases involved in signal transduction (CDG00002586 and CDG00002587 in QTL6.1). The later one showed a positive fold change of 25.37 compared to the untreated control. This gene is also located between our best SNP marker on QTL6.1 (Tcm006s19715703) and the closest neighboring SNP (Tcm006s19387431). Three other genes, CGD0026033 in QTL6.2, CDG0031650 and CDG0031682 in QTL9.2, are involved in photosynthetic, carbon and nitrogen metabolism, more specifically in sugar transport, beta-oxidation of fatty acids and amino acid metabolism, respectively [77]. It is interesting that many of these identified genes are actually very close to the most significant SNPs in each QTL identified in our study (Additional file 13). It would be of interest as well to investigate all these genes identified by Teixeira et al. [77] during the very early stages of infection.

## Conclusions

One of the main requirements of an efficient MAS program in cacao is to have reliable markers associated with the trait(s) of interest. These markers and ultimate knowledge of the underlying genes must be identified first. This knowledge demands a combination of large mapping populations with very distinct parents that segregate for the trait(s) of interest, a dense set of mapped markers, and rigorous phenotyping capabilities. The availability of a sequenced genome helps in the identification and annotation of potential candidate genes, although it is important to mention here that annotations are based on the Matina 1–6 genome, which is susceptible to WBD. It is therefore not unlikely that one or more genes responsible for the resistance phenotype occur specifically in the resistant cultivars, which might render the use of the Matina 1–6 genome inappropriate. We combined these requirements to identify several genomic regions that are associated with WBD resistance, and a novel source of resistance that most likely comes from the ‘CCN 51’ parent. Thanks to the large population size of the MP01 population, we were able to pick up QTL and markers with relatively small effects that can contribute to the creation and selection of more tolerant/resistant plant material.

## Additional files

Additional file 1:
**Layout of the field experiment. Individual trees were planted in 2002 in a 3 × 3 m grid.** Every fifth row in the field contained 5–19 trees (depending on the length of the rows) of the variety ‘Comum’, which is susceptible to WBD and serves as a natural and permanent inoculum source. Shade was provided using the traditional ‘cabruca’ system in which the trees are grown amongst the Atlantic Forest's native canopies. (XLS 97 kb) Additional file 2:
**Methods – Differential gene expression analysis.** (DOC 70 kb) Additional file 3:
**Primer and probe sequences for candidate gene expression analysis.** (XLS 35 kb) Additional file 4:
**Comparison of the linkage maps based on markers of chromosome IX with segregation types ab × aa (IX_ab × aa), ab × ab (IX_ab × ab) and aa × ab (IX_aa × ab), and the integrated linkage map (IX) of chromosome IX.** (DOC 62 kb) Additional file 5:
**Location of the SNP markers on the integrated linkage map.** (XLS 278 kb) Additional file 6:
**Segregation ratios of SNPs of type ‘ab × aa’ and of ‘aa × ab’.** Segregation ratios in the maternal and paternal meioses as observed in markers with segregation types ab × aa (blue) and aa × ab (red) plotted against their position on the integrated linkage map. The segregation ratio represents the proportion of alleles from the first grandparent, as determined by the phase of the SNP. (DOC 161 kb) Additional file 7:
**Positions of SNPs on the integrated linkage map (in cM on the** **y-axis** **) plotted against their corresponding positions on the physical map (in Mbp on the** **x-axis).** (DOC 887 kb) Additional file 8:
**Phenotypic data of witches’ broom disease in MP01.** (XLS 184 kb) Additional file 9:
**Phenotypic evaluation of witches’ broom disease resistance.** **(a)** Distribution of total numbers of vegetative brooms (VB) and cushion brooms (CB) over the period 2008 – 2011. The numbers on the X-axis represent the different bins with the number of brooms. For both traits, the distributions were highly skewed and contained many zeros. **(b)** Number of years in which a tree carried VB and the number of years in which it carried CB. **(c)** Adjustment of the data for row and column effects for possible spatial patterns of infestation. (DOC 100 kb) Additional file 10:
**Graphical results of the simulation study.** The results are obtained using 500 random samples from the original set of 459 individuals. ‘%hit per position’ denotes the average percentage of samples in which a marker position is selected as a QTL. (DOC 72 kb) Additional file 11:
**P-values of single and multiple QTLs and haplotype combinations associated with witches’ broom disease.** The first tab contains the single QTL with their different parental haplotype combinations. The different columns contain the total number of trees with each haplotype and the results of the Chi-squared test for resistance and susceptibility. The last column contains the percentage of resistant trees with the particular haplotype combination. The second and third tab contain the same info but for combinations of two and three QTL, respectively. The combinations marked in grey belong to the top five of lowest P-values and indicate the highest associations between the QTL and WBD resistance/susceptibility. (XLS 81 kb) Additional file 12:
**Haplotype analysis of trees exhibiting recombination in the QTL9.**1 segment on chromosome IX, associated with witches’ broom resistance. The maternal (‘TSH 1188’) and paternal (‘CCN 51’) haplotypes, as defined by iXora, are shown on top of the figure, together with the SNP markers. Alleles in grey represent alleles for which the haplotype could not be assigned by iXora due to the presence of the same allele in the other copy. The recombinant trees are presented with the number of total vegetative brooms (TVB) over the period of 4 years (more than 10 TVB equals susceptible trees). The first set of trees are resistant and all of them have the T2 haplotype coming for ‘TSH 1188’. The trees indicated in orange are resistant and do not contain the T2 haplotype, however, they do contain favorable alleles of some of the other QTL. The trees indicated in red are susceptible and do not contain the G-allele, neither any of the other favourable alleles. (DOC 63 kb) Additional file 13:
**Genes identified in all of the QTL regions associated with witches’ broom disease resistance.** The annotation of the genes was based on the Matina 1–6 v1.1. genome. The genes indicated in red are genes that are most likely involved in disease resistance. Genes indicated in yellow contain a SNP marker that had the highest association with the disease in each QTL. Genes indicated in orange were identified as differentially expressed by Teixeira et al. [77]. The homologues in *Arabidopsis thaliana* (if known) were used, together with the GO Slim terms to identify the cellular components, the molecular components and the biological functions of the genes within the QTL regions. (XLS 155 kb) Additional file 14:
**GO Slim annotation of gene models in the QTL regions.** The gene models from the Matina 1–6 assembly were screened and GO Slim annotation was used to classify the gene models according to their biological process. The X-axis shows the total number of gene models in all the QTL regions. The percentage after the bar represents the percentage of gene models within each annotated group (DOC 73 kb) Additional file 15:
**Results and discussion – Differential gene expression analysis.** (DOC 49 kb) Additional file 16:
**Differential gene expression analysis of candidate genes.** (XLS 300 kb)
