# Supplementary material for: Identification of candidate genes involved in Witches’ broom disease resistance in a segregating mapping population of Theobroma cacao L. in Brazil
Source: BMC Genomics. 2016 Feb 11;17:107. doi: 10.1186/s12864-016-2415-x (PMC4750280; doi:10.1186/s12864-016-2415-x)
Supplement: Additional file 2: — Methods – Differential gene expression analysis. (DOC 70 kb) [file 12864_2016_2415_MOESM2_ESM.doc]

**Additional file 2 Methods - Gene expression analysis**

**Plant and fungal material**

The plants used for artificial infection with *M. perniciosa* were ‘TSH 1188’ and ‘CCN 51’ plants that were obtained via budwood grafted onto ‘TSH1188’ rootstock and ‘TSH 1188’, ‘CCN 51’ and ‘CATONGO’ seedlings. The seedling plants were approximately one month old, whereas the grafted material was approximately one year old. Ten seedlings of each of the three genotypes were used as controls for the inoculation and kept in a greenhouse (controlled temperature and humidity) for 60 days or until the symptoms of WBD appeared, mainly for the moderately resistant/tolerant ‘CCN 51’ and the susceptible ‘CATONGO’. One group of grafted plants was mock inoculated (three apical meristems per plant), whereas the other group was inoculated with the fungus. After inoculation, a plastic bag containing wet cotton balls was wrapped around the plants of the grafted material to maintain humidity until the apical meristems were collected. For the seedlings, the bag was removed after 48 hours, and the plants were kept in a controlled greenhouse. The inoculated apical meristems of the controls at zero hours were collected, as well as at three, six, 12, 48 and 72 hours after inoculation. For each time point, three biological replicates were collected for both the mock inoculation and for the inoculation with the fungus.

The fungus that was used was obtained from dry witches’ brooms that were collected at MCCS. The spores were collected, and the inoculum was produced according to Frias et al. The strain of the fungus was identified as *Moniliophthora perniciosa* MARS/MCCS. The final concentration of the spore solution was 1×10^5^ basidiospores per milliliter, and 30 µL of the inoculum was used on each apical meristem.

**RNA extraction and cDNA synthesis**

Following a modified form of the methods described by Chang et al. and Verica et al. RNA was extracted from developing meristems collected at various hours after infection with inoculate or water. The apical meristems (with small leaves and an average size of 1 cm) were collected from each tree and placed immediately in liquid nitrogen. They were then ground to a fine powder and homogenized in 15 mL of 65°C extraction buffer using a PRO200 Homogenizer with a 10×115-mm generator (PRO Scientific, Inc., CT, USA). All of the samples were extracted twice with equal volumes of chloroform (1-min homogenization with the PRO200 Homogenizer and 30-min centrifugation at 10,500×g). One-third volume of 8 M LiCl was then added, the samples were mixed, and the RNA was precipitated overnight or over the weekend at 0°C (in an ice water bath in a 4°C refrigerator). The RNA pellet was obtained by centrifugation at 10,500×g for 1 hour at 4°C. The pellet was then processed with the Qiagen RNeasy Mini Kit (Qiagen, CA, USA) following the manufacturer’s instructions. The clean RNA was then treated with DNase using the RQ1 RNase-free DNase kit (Promega). The RNA treated with DNase was cleaned using phenol/chloroform/isoamyl alcohol and centrifuged for 10 min at 10,500×g at 4°C. The supernatant was collected, and 20 µL of 3 M NaOAc and 600 µL of 100% ethanol were added and stored overnight at -80°C. The solution was then centrifuged for 30 min at 10,500×g at 4°C. The pellet was washed with 80% ethanol, centrifuged for 30 min at 10,500×g at 4°C, dried, and resuspended with 50 µL of RNase-free water. The RNA was quantified using the spectrophotometer GeneQuant (GeneQuant^TM^ Pro RNA/DNA calculator, GE Healthcare Biosciences). First-strand cDNA synthesis was carried out in 20 µL reactions using the SuperScript® VILO™ cDNA Synthesis Kit (Invitrogen) according to the manufacturer’s instructions.

**Preparation of qPCR reactions according to the Fluidigm® 192.24 SNPtype™ Genotyping protocol**

Due to the small reaction volumes that are used by the Fluidigm system, a pre-amplification step is recommended to ensure that sufficient copies of the template are present for detection. This Specific Target Amplification (STA) reaction is performed prior to qPCR using either the same primers or primers flanking the qPCR primers. The STA reactions were set up according to the protocol that was provided by the manufacturer (Fluidigm). In brief, all of the STA primers were pooled together and diluted in DNA Suspension Buffer (10 mM Tris, pH 8.0, 0.1 mM EDTA) so that each primer had a final concentration of 180 nM. The pooled STA primers (1.25 µL), PreAmp Master Mix (1 µL; Fluidigm), and water (1 µL) were combined with cDNA (1.25 µL) for each examined sample, vortexed, centrifuged, and thermocycled (95°C for 2 min, followed by 14 cycles of 95°C for 15 seconds and 60°C for 4 min). The resulting pre-amplified products were then diluted 1:5 by the addition of 20 µL of TE Buffer (10 mM Tris-HCl, 1 mM EDTA). Next, the reactions were prepared for real-time PCR on the Fluidigm BioMark (Fluidigm). The assay mixtures were prepared by diluting the primers and TaqMan probes in DNA Suspension Buffer to final concentrations of 9 µM and 2 µM, respectively. The samples were prepared according to standard methods (1.5 µL of TaqMan Universal PCR Master Mix (2×) (Life Technologies), 0.15 µL (20×) of GE Sample Loading Reagent (Fluidigm), and 1.35 µL of diluted pre-amplified cDNA). All of the assay and sample mixtures were vortexed, centrifuged and loaded onto a 192.24 Gene Expression chip. After chip loading, real-time PCR was performed on the BioMark using the GE 192×24 standard v1 cycling parameters (50°C for 2 min and 95°C for 10 min, followed by 40 cycles of 95°C for 15 seconds and 60°C for 1 min). The data were collected and analyzed with Fluidigm Real-Time PCR Analysis 4.12 software (Fluidigm).

**Reference housekeeping genes and candidate genes**

Three reference genes (actin (Thecc1EG041851), elongation factor-1α (EF1α, Thecc1EG043087) and malate dehydrogenase (MDH, Thecc1EG020890)) were used for normalization (**Additional file 3**). The sequences of the candidate genes were obtained from the Matina 1-6 transcripts . Intron-exon splice junctions were identified, and TaqMan probes were designed to be transected by splice junctions to prevent the amplification of any possible contaminating genomic DNA. TaqMan primers and probes and flanking STA primers for pre-amplification were designed using the Primer Express 3.0 software (ABI). The primer and probe sequences are presented in **Additional file 3**.

**Determination of relative expression**

Each time point had three biological and three technical replicates. The ‘CCN 51’ samples were quantified relative to the mock-inoculated ‘CCN 51’ control at zero hours, whereas the ‘TSH 1188’ samples were quantified relative to the mock-inoculated ‘TSH 1188’ control at zero hours. The results were normalized using the Ct values that were obtained for the three endogenous reference genes that were present in the same reaction. First, the mean of the first biological replicate was calculated using the three technical replicates, as were the means for the second and third biological replicates. From these three means, the overall Ct mean for the particular sample was derived together with its standard deviation. The normalization was performed using the following equation: ΔCt = Ct_(target gene)_– Ct_(endogenous reference gene)_. The calibration was determined by the formula ΔΔCt = ΔCt_(sample)_–ΔCt_(calibrator)_. The relative quantification was obtained by the formula 2^–ΔΔCt^ . The best endogenous reference gene was determined with the RefFinder tool , which integrates the currently available major computational programs (geNorm , Normfinder , BestKeeper , and the comparative ΔCt method ) to compare and rank the tested candidate reference genes. Based on the rankings from each program, it assigns an appropriate weight to an individual gene and calculates the geometric mean of the weights for the overall final ranking .

**References**

1. Frias GA. An inoculation method for evaluating resistance of cacao to Crinipellis perniciosa. Plant Dis. 1995;79(8):787. doi:10.1094/pd-79-0787.

2. Chang S, Puryear J, Cairney J. A simple and efficient method for isolating RNA from pine trees. Plant Mol Biol Rep. 1993;11(2):113-6. doi:10.1007/bf02670468.

3. Verica JA, Maximova SN, Strem MD, Carlson JE, Bailey BA, Guiltinan MJ. Isolation of ESTs from cacao (Theobroma cacao L.) leaves treated with inducers of the defense response. Plant Cell Rep. 2004;23(6):404-13. doi:10.1007/s00299-004-0852-5.

4. Bailey BA, Strem MD, Bae HH, de Mayolo GA, Guiltinan MJ. Gene expression in leaves of Theobroma cacao in response to mechanical wounding, ethylene, and/or methyl jasmonate. Plant Sci. 2005;168(5):1247-58. doi:DOI 10.1016/j.plantsci.2005.01.002.

5. Motamayor JC, Mockaitis K, Schmutz J, Haiminen N, Livingstone D, Cornejo O et al. The genome sequence of the most widely cultivated cacao type and its use to identify candidate genes regulating pod color. Genome Biol. 2013;14(6):R53.

6. Livak KJ, Schmittgen TD. Analysis of relative gene expression data using real-time quantitative PCR and the 2^-ΔΔCT Method. Methods. 2001;25(4):402-8. doi:10.1006/meth.2001.1262.

7. Schmittgen TD, Livak KJ. Analyzing real-time PCR data by the comparative CT method. Nat Protoc. 2008;3(6):1101-8. doi:10.1038/nprot.2008.73.

8. Xie F, Xiao P, Chen D, Xu L, Zhang B. miRDeepFinder: a miRNA analysis tool for deep sequencing of plant small RNAs. Plant Mol Biol. 2012;80(1):75-84. doi:10.1007/s11103-012-9885-2.

9. Vandesompele J, De Preter K, Pattyn F, Poppe B, Van Roy N, De Paepe A et al. Accurate normalization of real-time quantitative RT-PCR data by geometric averaging of multiple internal control genes. Genome Biol. 2002;3(7):RESEARCH0034.

10. Andersen CL, Jensen JL, Orntoft TF. Normalization of real-time quantitative reverse transcription-PCR data: a model-based variance estimation approach to identify genes suited for normalization, applied to bladder and colon cancer data sets. Cancer Res. 2004;64(15):5245-50. doi:10.1158/0008-5472.CAN-04-0496.

11. Pfaffl MW, Tichopad A, Prgomet C, Neuvians TP. Determination of stable housekeeping genes, differentially regulated target genes and sample integrity: BestKeeper – Excel-based tool using pair-wise correlations. Biotechnol Lett. 2004;26(6):509-15. doi:10.1023/b:bile.0000019559.84305.47.

12. Silver N, Best S, Jiang J, Thein S. Selection of housekeeping genes for gene expression studies in human reticulocytes using real-time PCR. BMC Mol Biol. 2006;7(1):33.
