# Supplementary material for: Identification of candidate genes involved in Witches’ broom disease resistance in a segregating mapping population of Theobroma cacao L. in Brazil
Source: BMC Genomics. 2016 Feb 11;17:107. doi: 10.1186/s12864-016-2415-x (PMC4750280; doi:10.1186/s12864-016-2415-x)
Supplement: Additional file 4: — Comparison of the linkage maps based on markers of chromosome IX with segregation types ab × aa (IX_ab × aa), ab × ab (IX_ab × ab) and aa × ab (IX_aa × ab), and the integrated linkage map (IX) of chromosome IX. (DOC 62 kb) [file 12864_2016_2415_MOESM4_ESM.doc]

**IX_abxaa IX IX_abxab IX IX_aaxab**
