# Supplementary figures and images for: Identification of candidate genes involved in Witches’ broom disease resistance in a segregating mapping population of Theobroma cacao L. in Brazil
Source: BMC Genomics. 2016 Feb 11;17:107. doi: 10.1186/s12864-016-2415-x (PMC4750280; doi:10.1186/s12864-016-2415-x)

**Additional file 6**


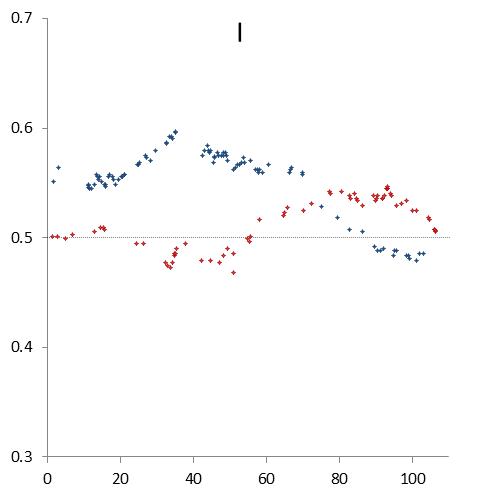

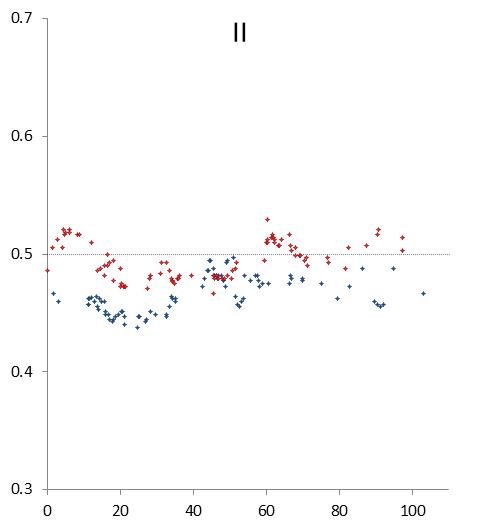

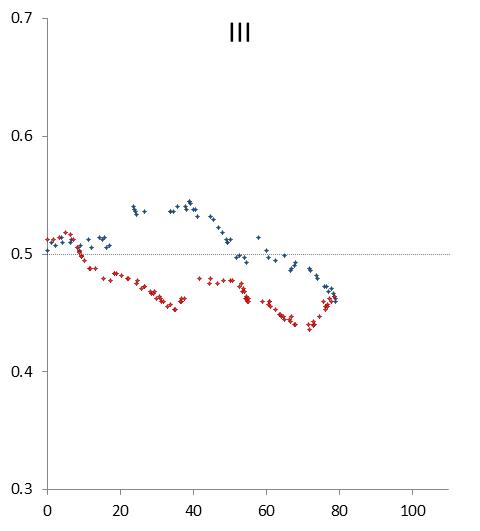

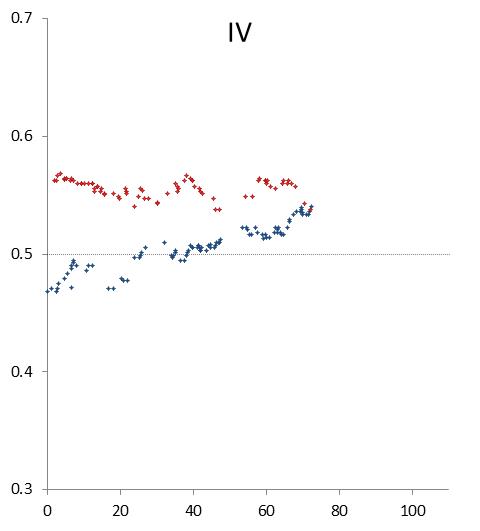

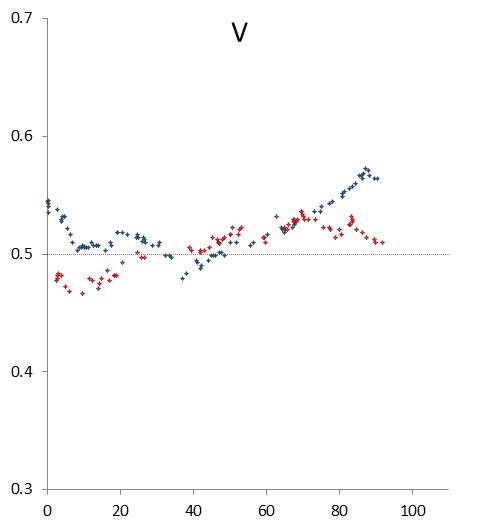

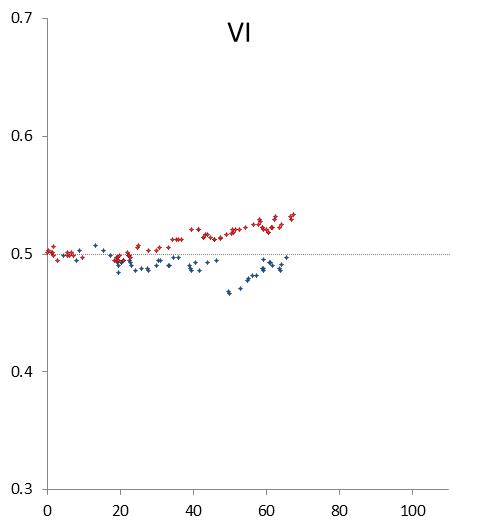

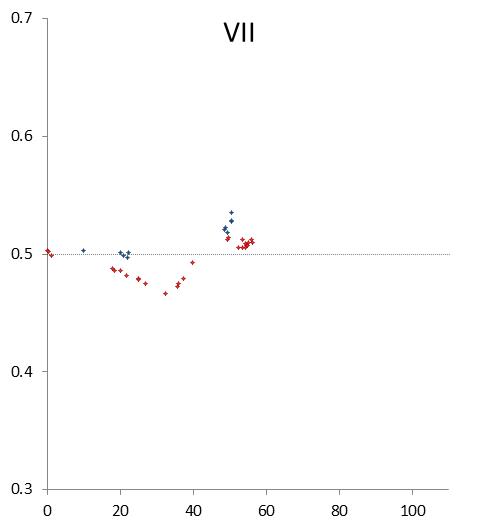

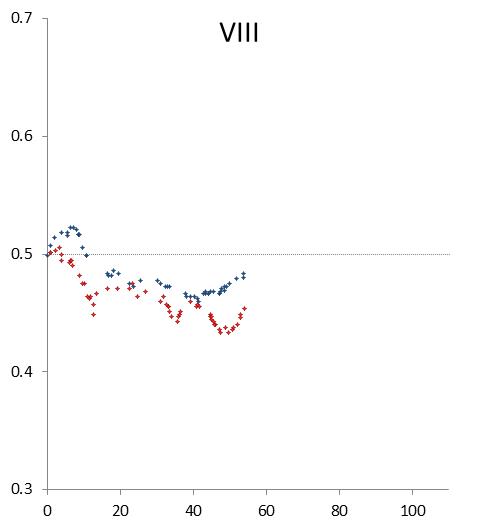

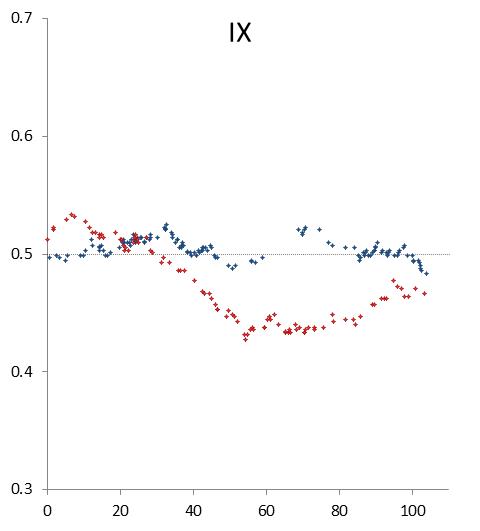

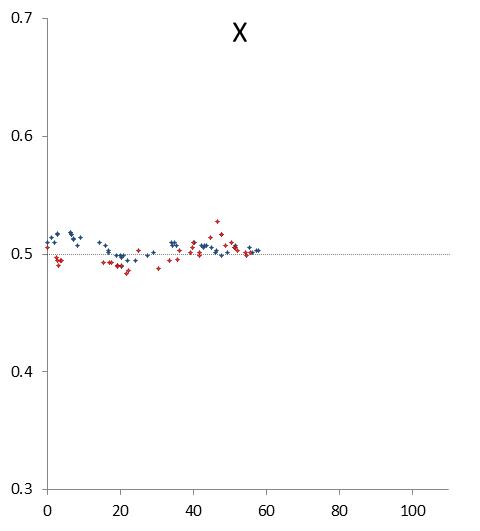

Supplement: Additional file 6: — Segregation ratios of SNPs of type ‘ab × aa’ and of ‘aa × ab’. Segregation ratios in the maternal and paternal meioses as observed in markers with segregation types ab × aa (blue) and aa × ab (red) plotted against their position on the integrated linkage map. The segregation ratio represents the proportion of alleles from the first grandparent, as determined by the phase of the SNP. (DOC 161 kb) [file 12864_2016_2415_MOESM6_ESM.doc]

**Additional file 9**

**(a)**


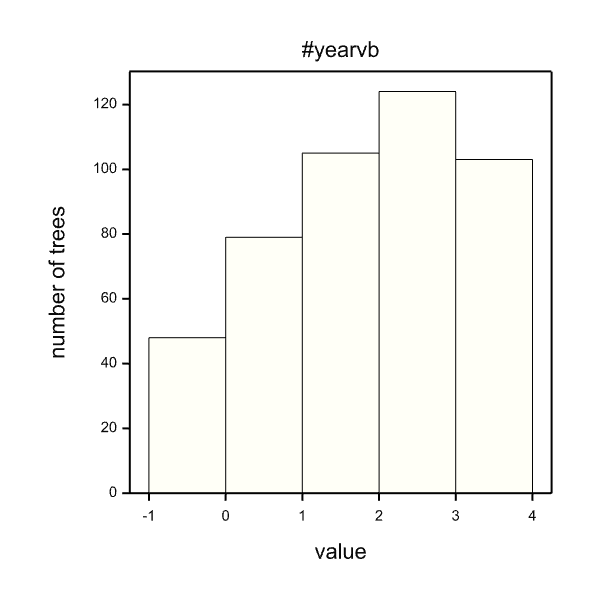

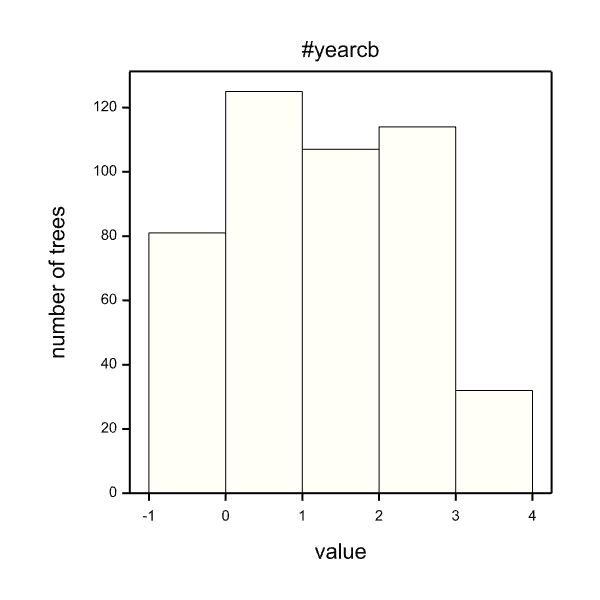

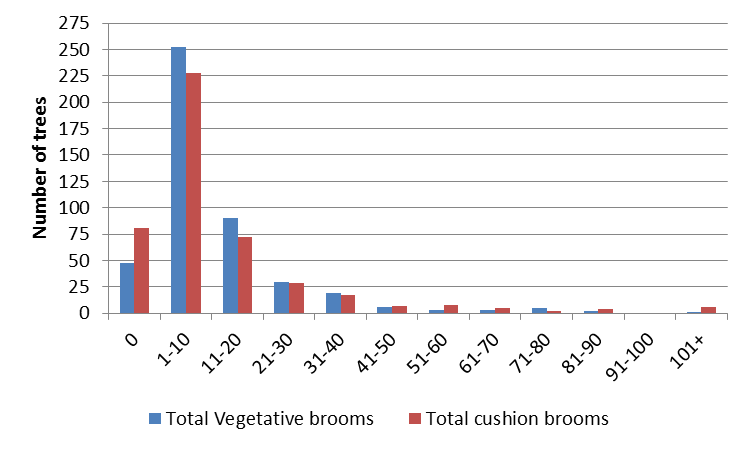


(**b**)


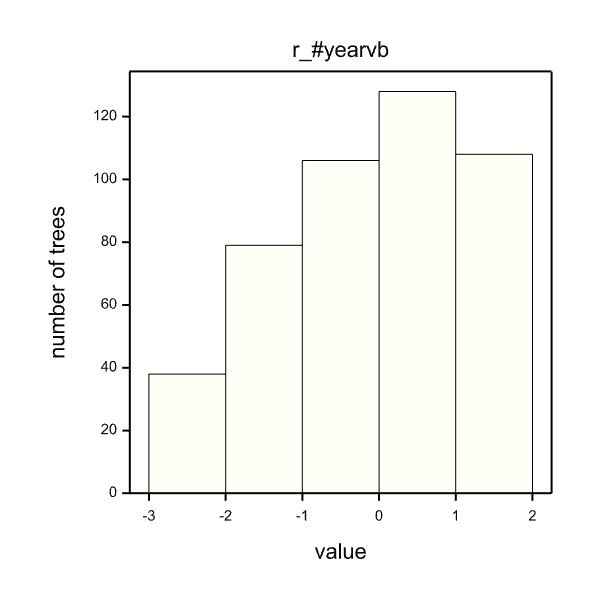

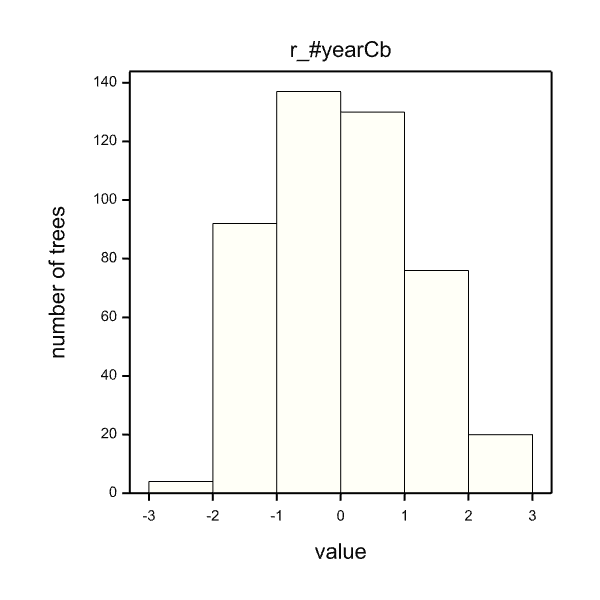


(**c**)

Supplement: Additional file 9: — Phenotypic evaluation of witches’ broom disease resistance. (a) Distribution of total numbers of vegetative brooms (VB) and cushion brooms (CB) over the period 2008 – 2011. The numbers on the X-axis represent the different bins with the number of brooms. For both traits, the distributions were highly skewed and contained many zeros. (b) Number of years in which a tree carried VB and the number of years in which it carried CB. (c) Adjustment of the data for row and column effects for possible spatial patterns of infestation. (DOC 100 kb) [file 12864_2016_2415_MOESM9_ESM.doc]

**Additional file 10**


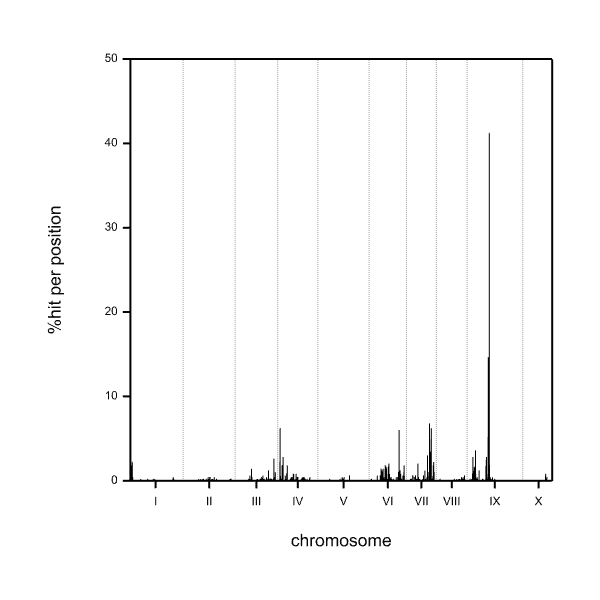

Supplement: Additional file 10: — Graphical results of the simulation study. The results are obtained using 500 random samples from the original set of 459 individuals. ‘%hit per position’ denotes the average percentage of samples in which a marker position is selected as a QTL. (DOC 72 kb) [file 12864_2016_2415_MOESM10_ESM.doc]

**Additional file 14**


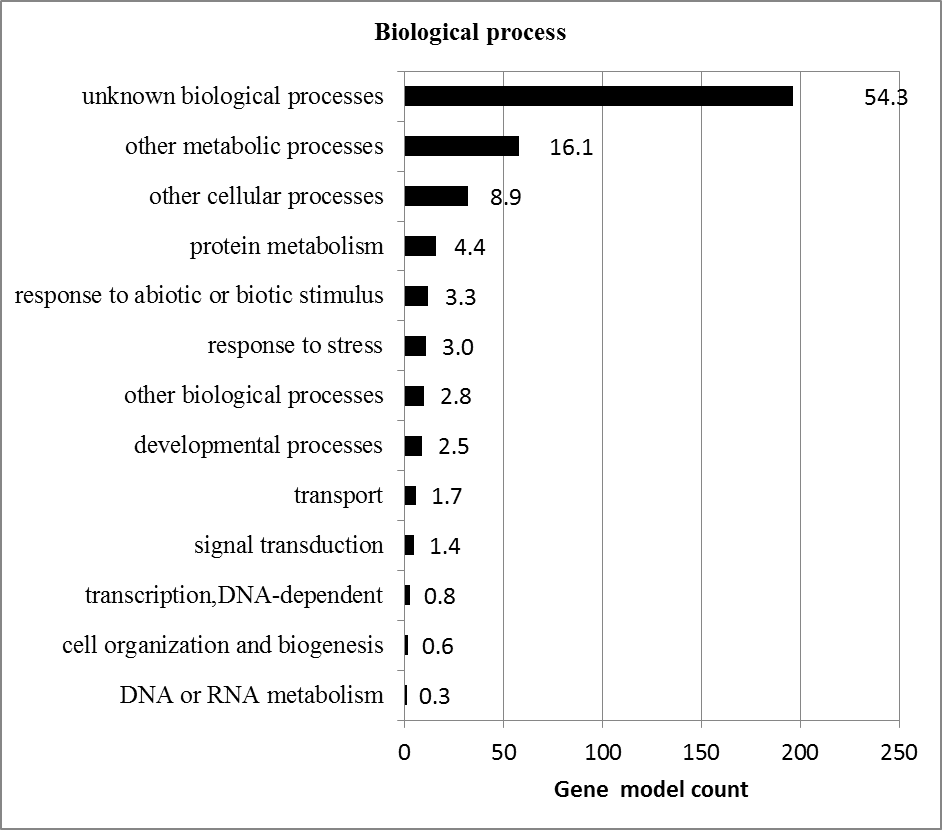

Supplement: Additional file 14: — GO Slim annotation of gene models in the QTL regions. The gene models from the Matina 1–6 assembly were screened and GO Slim annotation was used to classify the gene models according to their biological process. The X-axis shows the total number of gene models in all the QTL regions. The percentage after the bar represents the percentage of gene models within each annotated group (DOC 73 kb) [file 12864_2016_2415_MOESM14_ESM.doc]
