# Supplementary material for: Identification of candidate genes involved in Witches’ broom disease resistance in a segregating mapping population of Theobroma cacao L. in Brazil
Source: BMC Genomics. 2016 Feb 11;17:107. doi: 10.1186/s12864-016-2415-x (PMC4750280; doi:10.1186/s12864-016-2415-x)
Supplement: Additional file 7: — Positions of SNPs on the integrated linkage map (in cM on the y-axis ) plotted against their corresponding positions on the physical map (in Mbp on the x-axis). (DOC 887 kb) [file 12864_2016_2415_MOESM7_ESM.doc]

**Additional file 7**
