# Supplementary material for: Identification of candidate genes involved in Witches’ broom disease resistance in a segregating mapping population of Theobroma cacao L. in Brazil
Source: BMC Genomics. 2016 Feb 11;17:107. doi: 10.1186/s12864-016-2415-x (PMC4750280; doi:10.1186/s12864-016-2415-x)
Supplement: Additional file 12: — Haplotype analysis of trees exhibiting recombination in the QTL9.1 segment on chromosome IX, associated with witches’ broom resistance. The maternal (‘TSH 1188’) and paternal (‘CCN 51’) haplotypes, as defined by iXora, are shown on top of the figure, together with the SNP markers. Alleles in grey represent alleles for which the haplotype could not be assigned by iXora due to the presence of the same allele in the other copy. The recombinant trees are presented with the number of total vegetative brooms (TVB) over the period of 4 years (more than 10 TVB equals susceptible trees). The first set of trees are resistant and all of them have the T2 haplotype coming for ‘TSH 1188’. The trees indicated in orange are resistant and do not contain the T2 haplotype, however, they do contain favorable alleles of some of the other QTL. The trees indicated in red are susceptible and do not contain the G-allele, neither any of the other favourable alleles. (DOC 63 kb) [file 12864_2016_2415_MOESM12_ESM.doc]

**Additional file 12**
