# Supplementary material for: Identification of candidate genes involved in Witches’ broom disease resistance in a segregating mapping population of Theobroma cacao L. in Brazil
Source: BMC Genomics. 2016 Feb 11;17:107. doi: 10.1186/s12864-016-2415-x (PMC4750280; doi:10.1186/s12864-016-2415-x)
Supplement: Additional file 15: — Results and discussion – Differential gene expression analysis. (DOC 49 kb) [file 12864_2016_2415_MOESM15_ESM.doc]

**Additional file 15 - Gene expression analysis – Results and discussion**

From the expression data, it seems that most of the candidate genes in the mock-infected ‘CCN 51’ have a stable basal expression only until 48 hours after infection (HAI), after which the expression decreases at 72 HAI for the genes Thecc1EG016564 (MAPKK 5), Thecc1EG028959 (leaf senescence-associated receptor-like protein kinase, putative isoform 2), Thecc1EG028960 (leucine-rich repeat protein kinase family protein, putative), Thecc1EG028968, and Thecc1EG038267 (kinase superfamily protein with an octicosapeptide/Phox/Bem1p domain, putative). Thecc1EG038261 (Uveal autoantigen with coiled-coil domains and ankyrin repeats) was highly upregulated between 3 and 48 HAI.

In the infected ‘CCN 51’, for most of the genes, the general pattern of gene expression seems to be a slight downregulation at 3 HAI (more extreme for Thecc1EG028960), followed by an upregulation at 6 HAI, and between 12 and 48 HAI, the expression levels stabilized to a basal level. An opposite expression pattern was seen for Thecc1EG038261, which still showed high expression at 3 and 12 HAI but with a sharp decrease at 6 HAI. Thecc1EG038262 (RING/U-box super family protein that is associated with our most significant SNP in QTL9.1), which has a stable basal level expression in the mock-infected ‘CCN 51’, was only upregulated (more than 2-fold) at 6 HAI in the infected ‘CCN 51’.

In the ‘TSH 1188’ mock-infected plants, the majority of the genes showed stable basal expression, with significant downregulation at approximately 12 HAI. Thecc1EG038261 had a higher expression level, similar to that of ‘CCN 51’, but it decreased to the basal level only at 12 HAI. Thecc1EG028959 and Thecc1EG028960 were downregulated at 3 HAI and continued to be downregulated until 72 HAI.

In the infected ‘TSH 1188’, approximately half of the candidate genes showed a stable basal level of expression. Four genes, namely Thecc1EG028959, Thecc1EG028960, Thecc1EG038261 and Thecc1EG038267, were downregulated at 3 HAI and remained at that expression level at 6 HAI. The former two genes were even more downregulated at 12 HAI, whereas Thecc1EG038267 increased to the basal level and Thecc1EG038261 was upregulated. Conversely, Thecc1EG016564 was downregulated at 12 HAI. From 48 to 72 HAI, most of the genes returned to their basal expression levels, whereas Thecc1EG038261 went from upregulated to downregulated, after which it stabilized. The two downregulated genes, Thecc1EG028959 and Thecc1EG028960, returned to their basal expression levels.

Of the 11 selected candidate genes, only six showed considerable expression differences between infected and mock-infected plants for both ‘CCN 51’ and ‘TSH 1188’. The most remarkable expression differences were seen for Thecc1EG038261. When comparing the infected ‘CCN 51’ plants, it was clear that this gene was approximately 4-fold upregulated at 3 and 12 HAI, whereas it has a basal level of expression at the other time points. In the resistant ‘TSH 1188’, this gene was approximately 8-fold downregulated at 3 HAI, whereas it had a similar expression profile for the other time points. Two other remarkable genes, Thecc1EG028959 and Thecc1EG028960 (both generally annotated as disease resistance genes), showed a basal level of expression at 12 HAI in ‘CCN 51’ but an approximately 16-fold downregulation in the resistant ‘TSH 1188’. This level of downregulation was similar for Thecc1EG028959 in the mock-infected ‘TSH 1188’ but was 4-fold for Thecc1EG028960.

A previous study that investigated the expression levels of candidate genes that were related to WBD used different time points . The expression of 23 candidate genes was analyzed at 48, 72, 120 and 240 hours after infection for one susceptible (‘ICS 39’) and two resistant genotypes (‘CAB 208’ and ‘CAB 214’). ‘ICS 39’ in general showed a smaller and later response (only at 120 and/or at 240 HAI), whereas ‘CAB 208’ and ‘CAB 214’ showed a quicker and more intense response at 48 and 72 HAI. In two other studies, the resistant ‘TSH 1188’ was also used, together with the susceptible ‘CATONGO’ in which inoculated meristems were harvested at 24, 48 and 72 HAI and 15 and 30 days after infection . In the second study, six genes that were differentially expressed in the two genotypes were used in RT-qPCR experiments. Four genes (“No-Hit”, RGC2, PPI, and HRGP) were upregulated in ‘TSH 1188’ during the early stages of infection (24 to 72 HAI), whereas two other genes (PR10, MRG) were upregulated in ‘CATONGO’ during the later stages (30 to 72 DAI) . Interestingly, according to recent histological observations, the germination of basidiospores occurred around 2 HAI in the susceptible ‘CATONGO’, and around 4 HAI in the resistant ‘TSH 1188’, but both reach their maximum at 6 HAI, and well-developed germination tubes were present at approximately 12 HAI . Penetration into the plant tissues was observed at 6 HAI for both susceptible and resistant genotypes, and in susceptible genotypes, primary hyphae were observed in the cortex under the epidermis at 48 HAI . This experiment will be followed by another experiment in which the most contrasting genotypes of MP01 will be inoculated with *M. perniciosa,* and gene expression will be analyzed for some of the candidate genes that may be involved in WBD.

**References**

1. Leal GA, Albuquerque PSB, Figueira A. Genes differentially expressed in Theobroma cacao associated with resistance to witches’ broom disease caused by Crinipellis perniciosa. Molecular plant pathology. 2007;8(3):279-92. doi:10.1111/j.1364-3703.2007.00393.x.

2. da Hora Junior BT, Poloni JdF, Lopes MA, Dias CV, Gramacho KP, Schuster I et al. Transcriptomics and systems biology analysis in identification of specific pathways involved in cacao resistance and susceptibility to witches' broom disease. Molecular bioSystems. 2012;8(5):1507-19. doi:10.1039/c2mb05421c.

3. Lopes MA, Hora BT, Jr., Dias CV, Santos GC, Gramacho KP, Cascardo JC et al. Expression analysis of transcription factors from the interaction between cacao and Moniliophthora perniciosa (Tricholomataceae). Genetics and molecular research : GMR. 2010;9(3):1279-97. doi:10.4238/vol9-3gmr825.

4. Sena K, Alemanno L, Gramacho KP. The infection process of Moniliophthora perniciosa in cacao. Plant Pathology. 2014;63(6):1272-81. doi:10.1111/ppa.12224.
